# High Physical Exposure During Female Recruits’ Basic Military Training in Sweden—A Descriptive Study

**DOI:** 10.1093/milmed/usad335

**Published:** 2023-08-25

**Authors:** Marie Kierkegaard, Matthias Tegern, Alexandra Halvarsson, Lisbet Broman, Helena Larsson

**Affiliations:** Department of Neurobiology, Care Sciences and Society, Division of Physiotherapy, Karolinska Institutet, Stockholm SE-141 83, Sweden; Academic Specialist Center, Center of Neurology, Stockholm Health Services, Stockholm SE-113 65, Sweden; Department of Neurobiology, Care Sciences and Society, Division of Physiotherapy, Karolinska Institutet, Stockholm SE-141 83, Sweden; Department of Community Medicine and Rehabilitation, Unit of Physiotherapy, Umeå University, Umeå SE-901 87, Sweden; Department of Neurobiology, Care Sciences and Society, Division of Physiotherapy, Karolinska Institutet, Stockholm SE-141 83, Sweden; Women’s Health and Allied Health Professionals Theme, Medical Unit Occupational therapy and Physiotherapy, Karolinska University Hospital, Stockholm SE-141 86, Sweden; Department of Neurobiology, Care Sciences and Society, Division of Physiotherapy, Karolinska Institutet, Stockholm SE-141 83, Sweden; Department of Neurobiology, Care Sciences and Society, Division of Physiotherapy, Karolinska Institutet, Stockholm SE-141 83, Sweden

## Abstract

**Introduction:**

There is a knowledge gap concerning the occurrence of physical complaints/injuries, i.e., musculoskeletal disorders (MSD), among Swedish women who undergo basic military training (BMT). The aims were to describe prevalence and factors related to MSD and explore physical exposure and performance in Swedish female recruits during BMT.

**Materials and Methods:**

A total of 144 females (mean age 22 years) who underwent BMT in 2016 participated in this cross-sectional study. Data regarding self-reported MSD, physical performance, physical activity and exercise, motivation and mental and physical preparation, and physical exposure during BMT and perceived health were collected at the end of BMT through the Musculoskeletal Screening Protocol questionnaire. Additional data on muscle strength were retrieved from IsoKai isokinetic lift tests. Descriptive and analytic (paired samples *t*-test and logistic binary regression) statistics were used.

**Results:**

The prevalence of MSD was high, with 33% (*n* = 48) reporting MSD before BMT, 78% (*n* = 113) during, and 50% (*n* = 72) at the end of BMT. Knee and upper back were the most frequently reported MSD locations. Forty-four (30%) participants felt insufficiently physically prepared for BMT. The physical exposure was high with loaded marches/runs and carrying heavy loads as the most demanding tasks. The longest walking distance was reportedly 55 km, and the reported maximum load was 50 kg. Forty-five participants (31%) had carried a load representing over 50% of their body weight. Most participants reported good to excellent health at the end of BMT. There was a small (8 N) but significant (*P* = 0.045) increase in mean force over time. Two variables, MSD before BMT (odds ratio 2.24, *P* = 0.03) and being physically unprepared (odds ratio 3.03, *P* < 0.01), were associated with MSD at the end of BMT.

**Conclusion:**

This study showed that the prevalence of MSD in Swedish female recruits was high before, during, and at the end of BMT, with knee and upper back as the most frequent locations. Although the physical exposure during BMT was occasionally high, self-rated health was mainly perceived as good to excellent at the end of BMT. Previous MSD and being physically unprepared were related to MSD at the end of BMT. These important and relevant findings indicate the necessity for implementing interventions to increase physical fitness and treat MSD at the beginning of BMT.

## INTRODUCTION

The integration of women into typically manly professions, such as armed forces, has been a conversional issue worldwide. In Sweden, the parliament decided in 1978 that women could be employed as commanders in the Swedish Armed Forces (SwAF), and in 1980 the first women reported for duty. In 1989, all military positions, including fighter pilot and submarine positions, were made available for women. It was, however, not until 1994 that the Swedish government abolished the requirement that women accepted for military service must train as officers. Whereas women had to apply for basic military training (BMT), it was compulsory for men from 1901 until 2010. The conscription system was then suspended in Sweden in favor of a recruitment system based on voluntary terms for both men and women. The Swedish government decided, however, for military readiness reasons, to re-activate conscription from January 1, 2018. Since 2018, recruitment into the SwAF is gender neutral and women perform their BMT together with men. Today, 22% of the approximately 23,600 persons in the SwAF are women. However, to meet set growth objectives with required competences, the number of women needs to increase. Thus, the proportion of women undergoing BMT must increase.

The Swedish BMT is a 3-month education of theory and practice, including physical exercise, field training, and military-specific training such as weapon and equipment handling, military drills, load carriage, and marching. Musculoskeletal injuries and complaints, i.e., musculoskeletal disorders (MSD), during BMT are common as shown by studies from the United States,^1–3^ Australia,^4^ New Zealand,^5^ and various European countries.^6–10^ In addition, there is an increased risk of premature discharge among recruits reporting MSD before^11^ or during BMT.^1–3,9^

Identified risk factors for MSD in the general military population include age,^3,12^ overweight and/or high body mass index (BMI),^12,13^ previous injuries,^12,13^ and poor cardiorespiratory and muscular fitness.^12,13^ In addition, studies show that long distance marching and load carriage are also associated with military soldier injuries.^14–16^ The literature exploring factors associated with MSD during BMT have come to approximately the same results. Thus, older age,^17^ height,^2^ low BMI,^6^ previous injuries,^2,6^ low physical activity and fitness (muscular and aerobic) levels prior to BMT,^10,17^ load carriage,^18^ and total distance travelled during BMT^19^ are factors related to MSD during BMT.

There are conflicting results concerning sex, e.g., one systematic review with meta-analysis reports that it is not associated with an increased risk for injury,^12^ whereas another identifies female sex as being a significant predictor of MSD.^13^ Nevertheless, it seems like women have a higher injury rate compared to men during BMT.^2,8,20^ Furthermore, military women report more mental health issues compared to men.^21^ Reviews focusing on female military personnel propose that previous injuries and low level of physical fitness are associated with an increased risk of MSD.^22,23^ The above reported findings are mainly based on studies from the United States and might therefore not apply to other military settings and populations. There is a specific knowledge gap concerning the situation among Swedish women undergoing BMT. Thus, it is important to detect MSD so that appropriate preventive and rehabilitative strategies can be undertaken and thereby the risk for premature discharge be reduced. Accordingly, the aims of the present study were to describe the prevalence of self-reported MSD and explore self-reported physical exposure, self-reported physical performance, and change in muscle strength among Swedish female recruits, in addition, to identify factors related to MSD at the end of BMT.

## METHODS

### Study Design

A descriptive cross-sectional design (with additional longitudinal data on muscle strength) was adopted to answer the study objectives.

### Study Setting and Participants

All female recruits (*n* = 144, no dropouts) undergoing BMT starting between May and October 2016 participated in the study. They were at the end of their BMT invited to one of the Swedish defense recruitment agencies, i.e., in Stockholm, Gothenburg, or Kristianstad, to re-assess isokinetic lift strength and answer the Musculoskeletal Screening Protocol (MSP) follow-up questionnaire. Results from their isokinetic lift strength tests performed at enrollment were retrieved from the recruitment agencies. All participants received written information and signed a written informed consent. The regional board of ethics in Stockholm granted approval for the study (Dnr 2016/2073-32), and procedures were conducted in accordance with the Declaration of Helsinki.

### Measures

The self-administered MSP follow-up questionnaire was used for data collection regarding age, height, weight, MSD, physical performance, physical activity and exercise, motivation and mental and physical preparation, physical exposure during BMT, and perceived health. The questionnaire is used to investigate prevalence of MSD and perceived health in military personnel^24–26^ and has been tested for reliability.^27^ Questions covered the following:

- Occurrence of MSD, i.e., physical complaints /injuries, before BMT with yes and no as response options, and occurrence of MSD during and at the end of BMT in 10 anatomical parts (neck, upper back, lower back, shoulder, elbow, hand, hip, knee, lower leg, and foot) with yes and no as response options, including whether these MSD had affected the ability to take part in the training. Dichotomized variables were constructed representing the occurrence of any MSD or none. The number of anatomical locations answered with a yes was summarized for each participant concerning MSD during and at the end of BMT.
- Intensity of MSD at the end of BMT rated on a 11-point numeric rating scale (NRS) ranging from 0 (not at all) to 10 (worst imaginable).
- Frequency of MSD during BMT rated as rarely, frequently, or all the time, and whether a physiotherapist, nurse, and/or medical doctor had been consulted for these complaints, with yes and no as response options.
- Physical performance, i.e., how the physical part of BMT regarding muscular strength and cardiorespiratory fitness had been managed, rated on a 4-point ordinal scale from very poorly to very well, which was dichotomized into well and poorly.
- Participant’s weekly amount of planned physical exercise as part of BMT, and cardiorespiratory fitness and/or muscle strength training on own initiative (number of times/week).
- Perceived change in cardiorespiratory fitness and muscle strength at the end of BMT, rated on a 4-point ordinal scale from a lot worse to a lot better, which was dichotomized into deteriorated and improved.
- Motivation for undergoing BMT and being mentally and physically prepared before BMT with yes and no as response options.
- Estimation of physical exposure during BMT, i.e., identification (open response option) of the most demanding task during BMT, which 10 anatomical parts were mainly loaded, and the frequency of this task, rated on a 4-point ordinal scale ranging from occasionally to daily. Participants’ identified most demanding task was classified into one of the following: carrying heavy loads, loaded marches/runs, or clinch/infighting. In addition, estimation of the longest march and carried weight during this march, with open response options in km and kg, respectively. Carried weight was also expressed as percentage of body weight (BW).
- Perceived insufficiency regarding physical performance during BMT with yes and no as response options, and if yes, which physical qualities (open response option) were perceived as insufficient.
- Perceived health, i.e., experience of physical and mental health, physical and social environment, and work ability, all rated on a 7-point NRS from 1 (very poor) to 7 (excellent), categorized into poor (≤3), good (4–5), and excellent (≥6).

The IsoKai isokinetic lift test was used to assess isokinetic force as a proxy for dynamic muscular strength. The test is described in detail in previous studies of validity and reliability^28,29^ and is used in the SwAF since 1995. In short, the isokinetic mean force in Newton (N) is registered from a force plate during the maximal two-handed lift of a weight-lifting bar from knee to shoulder level in the IsoKai machine which regulate the speed of the lift at 0.3 m/s. The starting position is standing on the force plate with feet shoulder width apart, the back straight while being forwardly inclined and knees bent. In line with the Swedish defense recruitment agencies procedures, participants were medically screened by a physician before being allowed to perform the test. Participants performed a 10-min warm-up session on a cycle ergometer and one submaximal practice test lift before performing two maximal lifts. There was no verbal encouragement during the test, and participants had 2–3 min rest between the two maximal lifts. The best of two trials were recorded.

### Statistical Analyses

Descriptive data were presented as number (*n*), percentage (%), mean with standard deviation (SD) for normally distributed data otherwise median with interquartile range (IQR), and minimum (min) and maximum (max) values. Decisions on normal distribution were based on calculations of skewness and kurtosis and the Shapiro-Wilk test. A paired samples *t*-test was used for the analysis of change in dynamic muscle strength (normally distributed data).

Logistic binary regression analyses were used to identify factors (independent variables) related to the dichotomized dependent variable MSD at the end of BMT. The selection of independent variables was based on previous research^2,6,10,17–19^ and clinical experience. Thus, the following variables were explored: age, anthropometric measures, previous MSD, dynamic muscle strength, mental and physical preparedness, perceived insufficient physical performance during BMT, longest marching distance, and load carriage. Univariate binary logistic regression analyses were performed, and independent variables showing an association (*P* < 0.20) with the dependent variable were included in a multivariable regression model. By using a stepwise backward deletion process, all non-significant (*P* ≥ 0.05) independent variables were removed, leaving only variables significantly associated with MSD at the end of BMT. Associations were reported as odds ratios (ORs) with corresponding 95% CI.

All analyses were performed using IBM SPSS Statistics version 28.0. A *P*-value <0.05 was considered statistically significant.

## RESULTS

### Descriptive Data

The 144 female recruits did their BMT in the following military branches: army (*n* = 84), air force (*n* = 37), medical center (*n* = 13), and navy (*n* = 10). Their mean (SD) age, height, weight, and BMI at end of BMT were 22 (3) years, 1.69 (0.06) m, 66.6 (7.0) kg, and 23.3 (2.0) kg/m2, respectively. There were in general few missing data concerning answers on the MSP follow-up questionnaire, at most from four participants. Twenty-four (17%) participants did not pass the medical examination at the end of their BMT due to either sickness or pain and were thus unable to perform the follow-up IsoKai isokinetic lift test.

Forty-eight (33%) participants reported perceived MSD before BMT. Out of these, 16 reported perceived deterioration of the MSD during BMT, while 13 reported perceived improvements. A total of 113 (78%) participants reported perceived MSD during BMT and 72 (50%) at the end of BMT. Ninety-three participants (82%) perceived that their MSD had affected their ability to take part in the training. Prevalence of self-reported MSD for all body regions during and at the end of BMT, and intensity ratings of perceived MSD at the end of BMT are presented in Table I. It was common to report MSD in more than one anatomical location. For example, recruits reported up to six different locations during BMT and up to five at the end of BMT (Table I). The most frequent locations of self-reported MSD both during and at the end of BMT were knee and upper back. Median intensity ratings at end of BMT were, however, highest for foot and neck MSD. Forty-five of the 113 recruits reporting MSD during BMT had these MSD frequently or all the time. As for consulting a medical professional, 69 recruits had consulted a physiotherapist, 51 a nurse, and 36 a medical doctor.

**TABLE I.** Prevalence of Musculoskeletal Disorders (MSD) during and at End of Basic Military Training (BMT), and Perceived Intensity of MSD at the End of BMT, *N* = 144

	Physical complaints/injuries				
	During BMT	At the end of BMT	Intensity, NRS		
Anatomical part	*n* (%)	*n* (%)	Median	IQR	Min–Max
Neck	11 (8)	6 (4)	6 ^#^	5–6	5–6
Upper back	34 (24)	18 (12.5)	5 *	4–6	2–8
Lower back	22 (15)	13 (9)	5 ^&^	3–5	1–8
Shoulder	33 (23)	16 (11)	3 ^&^	3–6	1–8
Elbow	5 (3.5)	4 (3)	3 ^#^	2–	2–5
Hand	20 (14)	12 (8)	2 *	1–4	0–5
Hip/buttock	17 (12)	5 (3.5)	4	3–6	2–6
Knee	43 (30)	21 (15)	4 *	3–6	1–7
Lower leg	16 (11)	11 (8)	5	4–6	3–7
Foot	31 (21.5)	17 (12)	6 ^#^	3–7	2–8
Number of anatomical parts	*n* (%)	*n* (%)			
Zero	31 (21.5)	72 (50)			
One	46 (32)	40 (28)			
Two	34 (24)	20 (14)			
Three	21 (14.5)	7 (5)			
Four	7 (5)	3 (2)			
Five	3 (2)	2 (1)			
Six	2 (1)				

BMT: basic military training, IQR: interquartile range, Min: minimum, Max: maximum, MSD: musculoskeletal disorders, Missing data from: *n = 1, ^#^*n* = 2, ^&^*n* = 3.

Number of recruits reporting MSD during and at the end of BMT, and perceived intensity of MSD at the end of BMT on a numeric rating scale (NRS) ranging from 0 (not at all) to 10 (worst imaginable). MSD could be reported in more than one anatomical part.

Almost all participants reported that they had managed the physical part of BMT well regarding muscular strength (92%) and cardiorespiratory fitness (82%, missing *n* = 1). Most reported participating in planned physical exercise 1–2 times/week (85%, missing *n* = 2), and 116 (81%) performed additional cardiorespiratory fitness and/or muscle strength training approximately twice weekly. While 117 participants (81%, missing *n* = 1) perceived improvements in cardiorespiratory fitness during BMT, 26 (18%) reported perceived deterioration. As for perceived change of muscle strength, 110 participants (76%, missing *n* = 3) reported an improvement and 31 (22%) a deterioration. All but six participants had been motivated to undergo BMT. Having felt sufficiently mentally and physically prepared for BMT was reported by 136 (92%) and 98 participants (68%, missing *n* = 2), respectively.

Reported physical exposure (*n* = 140), i.e., the identified most demanding task during BMT, was classified as carrying heavy loads for 83 (59%) participants, loaded marches/runs for 53 (38%), and clinch/infighting for 4 (3%) participants. These tasks were reported to occur occasionally for 103 (74%) participants and weekly or more for 37 (26%). Body parts that ≥70% of participants reported as mainly loaded were shoulders and knees for carrying heavy loads; and neck, upper back, shoulders, and knees for loaded marches and runs. The median (IQR, min–max) longest march distance was 28 (20–35, 3–55) km, and the median (IQR, min–max) carried load was 30 (25–35, 10–50) kg. The reported physical load was high, especially in relation to BW with 45 participants (31%) reported having carried a load representing over 50% of their BW during the longest march. Eighty-eight (61%) participants reported having had insufficient physical performance in performing tasks in a good way during BMT, and muscle strength, especially in arms and upper body, was the most common lacking physical quality.

**FIGURE 1.**
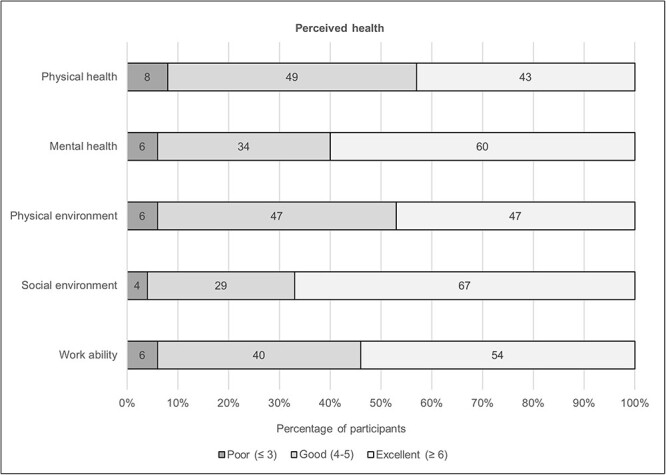
Perceived health categorized as poor, good, and excellent, *n* = 141 except for physical environment where *n* = 140.

Most participants reported good to excellent health at the end of BMT (Fig. 1). The median (IQR) was 5 (5–6) for both physical health and physical environment and 6 (5–6) for all others, i.e., mental health, social environment, and work ability. The min and max value for all health components was 2 (poor) and 7 (excellent, could not be better), respectively.

Results from the IsoKai isokinetic lift tests showed that the mean (SD) dynamic muscle strength was 589 (64) *N* (*n* = 144) and 588 (66) *N* (*n* = 120) at enrollment and 596 (67) *N* at the end of BMT (*n* = 120).

### Analyses

The mean (95% CI) difference in dynamic muscular strength for those able to perform the IsoKai isokinetic lift tests at both enrollment and end of BMT (*n* = 120) was 8 (0; 16) *N*, representing a small but significant (*P* = 0.045) increase in mean force over time. If expressed in relative values, the mean (SD) percentage change was 2 (8) %.

The univariate binary logistic regression analyses showed that five variables (MSD before BMT, mentally and physical preparedness before BMT, and perceived insufficient physical performance and longest march distance during BMT) were associated (*P* < 0.20) with the dependent variable (Table II) and thus included in a multivariable regression model (Table III). Two variables, MSD before BMT (OR 2.24, *P* = 0.03) and not being physically prepared (OR 3.03, *P* < 0.01), remained associated with MSD at the end of BMT in the final model (Table III).

**TABLE II.** Univariate Binary Logistic Regression Analyses

		MSD at the end of BMT		
	*n*	OR	95% CI	*P*-value
Age, years (cont.)	144	1.06	0.95–1.19	0.29
Height, cm (cont.)	144	0.98	0.94–1.03	0.35
Weight, kg (cont.)	144	0.98	0.94–1.03	0.39
BMI, kg/m^2^ (cont.)	144	0.97	0.83–1.14	0.75
Muscle strength, *N* (cont.)	144	1.00	0.99–1.00	0.58
MSD before BMT				
Yes	48	**2.14**	1.05–4.36	0.04
No	96		(ref)	
Mentally preparedness				
No	12	**3.23**	0.85–12.68	0.08
Yes	132		(ref)	
Physical preparedness				
No	44	**2.98**	1.41–6.31	<0.01
Yes	98		(ref)	
Perceived insufficient physical performance during BMT				
Yes	88	**1.75**	0.88–3.47	0.11
No	54		(ref)	
Longest march, km (cont.)	144	**0.98**	0.94–1.01	0.16
Carried weight				
kg (cont.)	144	1.01	0.97–1.06	0.61
Percentage of BW (cont.)	144	1.01	0.98–1.04	0.38

MSD: musculoskeletal disorders, BMT: basic military training, cont.: continuous variable, OR: odds ratios, CI: confidence interval, BMI: body mass index, BW: body weight.

Numbers in bold indicate independent variables that are significantly associated (*P* < 0.20) with the dependent variable MSD at the end of BMT and, thus, carried forward to the following multivariable regression model.

**TABLE III.** Initial and Final Multivariable Regression Model

		Musculoskeletal disorders at end of BMT					
		Initial model			Final model		
	*n*	OR	95% CI	*P*-value	OR	95% CI	*P*-value
MSD before BMT							
Yes	48	**2.78**	1.28–6.14	0.01	**2.24**	1.07–4.72	0.03
No	96						
Mentally preparedness							
No	12	2.57	0.59–11.21	0.21			
Yes	132						
Physical preparedness							
No	44	**2.51**	1.11–5.72	0.03	**3.03**	1.41–6.50	<0.01
Yes	98						
Perceived insufficient physical performance during BMT							
Yes	88	1.43	0.67–3.08	0.36			
No	54						
Longest march, km (cont.)	144	0.96	0.92–1.00	0.05			

MSD: musculoskeletal disorders, BMT: basic military training, OR: odds ratios, CI: confidence interval, cont.: continuous variable.

Numbers in bold indicate independent variables that are significantly associated (*P* < 0.05) with the dependent variable MSD at end of BMT.

## DISCUSSION

This is, to our knowledge, the first study exploring physical exposure and prevalence of MSD in women undergoing Swedish BMT. Even though self-reported health was rated as good to excellent, the findings of a high prevalence of MSD, heavy load carriage, and that previous MSD and not being physically prepared before BMT were associated with MSD at the end of BMT indicate the necessity for appropriate preventive measures.

The prevalence of MSD was surprisingly high, both during and at the end of BMT. The facts that 78% of the participants experienced at least one MSD during BMT, it affected their ability to take part in the training, and only 50% of participants were without any MSD at the end of BMT imply an unhealthy physical exposure. Previous studies using the MSP questionnaire have shown that 47% of 325 Swedish soldiers (8% women) report MSD during deployment and 28% at the end.^24^ Studies also reveal that the prevalence of MSD in Swedish soldiers (in total 961, 7% women) preparing for deployment has increased over time, i.e., from 7% in 2002 to 35% in 2012.^25^ These prevalence figures are considerably lower than those in our present study. This might be explained by the sex difference found in military personnel, where women report a higher rate of injuries compared to men.^13,20^ It could also be that MSD are more common in recruits, e.g., the prevalence of MSD is reportedly higher in female recruits compared to active female soldiers.^23^ Several systematic reviews show that MSD are common in both recruits undergoing BMT and in active soldiers.^12,13,17,20^ A recently published scoping review summarizing the literature on MSD among women in the military conclude that the prevalence of MSD in female recruits is high, with figures varying from 20% to 58%.^23^ Thus, our finding of a 78% prevalence of MSD during BMT raises concerns. However, the discrepancy in numbers between our study and others might be due to different definitions of MSD.

The knee was the most frequently reported location of MSD both during and at the end of BMT. This is in line with previous research on Swedish soldiers^24,26^ and recruits from the United States,^1^ Australia,^4^ New Zealand,^5^ and various European countries,^6,8–10^ where lower extremity MSD are reported to be most common. Other common sites for MSD among military personnel are the upper and lower back, as seen in the present study, and many others.^5,7,10,15,24,26^ These body parts were also those which participants reported to be mainly loaded during the most demanding tasks during BMT, i.e., carrying heavy loads and loaded marches and runs.

Although high physical exposure mostly occurred occasionally, about a quarter of our participants carried heavy loads and/or performed loaded marches/runs once a week or more often. Injuries attributed to load carriage and long-distance marching, i.e., overuse injuries, constitute a significant portion of the overall injury rate in soldiers and recruits undergoing BMT.^3,14–16^ Historically, the load carried by soldiers has increased, and today combat soldiers can carry as much as 60 kg.^30^ It is well known that heavy loads increase the physiological strain and reduce physical performance.^31^ Importantly, these issues are related to body size where smaller individuals, regardless of sex, are more affected.^31^ Thus, in addition to guidance about maximum loads in kg, recommendations on load carriage are also given in percentage of BW. Present guidelines state that the optimal load for combat missions is 20–30% of a soldier’s BW, and that for sustained non-contact movements load carriage should not exceed 45% of BW.^32^ However, carrying a load exceeding 25% of one’s BW is found to be associated with road marching–related injuries.^14^ In light of this finding and these recommendations, our result, with a third reporting a load carriage ≥50% of their BW, is worrying, especially since this is during the first 12 weeks of their military training and not during military operations.

In general, Swedish soldiers report good-to-excellent health both before and after deployment.^24,25^ This finding was corroborated among our participants at the end of BMT, which might seem contradictory considering the high prevalence of MSD and the unhealthy physical exposure. The median values for physical health and physical environment were, however, lower (indicating poorer health) than for the other categories. Although not shown in the present study, less than excellent self-reported physical health has previously been associated with both neck and back MSD in Swedish soldiers.^26^ Thus, there are reasons for addressing the physical exposure to which Swedish recruits and soldiers are exposed.

Muscle strength is a critical key physical component for military personnel and is generally reported to increase after BMT.^33^ Even though our observed mean change of 8 N (2%) was statistically significant, it cannot be seen as clinically relevant as it lies within the measurement error for the Isokai-test.^28^ It is surprising that the recruits in our study did not increase their dynamic muscular strength more as they participated in both planned exercise and additional muscle strength training twice weekly. That almost a fifth reported a deterioration in muscle strength and about two-thirds lacked the strength in arms and upper body to perform tasks in a good way indicates the need for specific physical training programs, especially since muscle strength and endurance are associated with an increased risk for MSD.^34^

The results from our multivariable regression model showed that the odds for MSD at the end of BMT were significantly higher for those women who had MSD before BMT and were physically unprepared. This is in line with results from systematic reviews where previous injuries and poor physical performance are found to be significant predictors of MSD in military personnel.^12,13,22^ Physical fitness level might be even more important for female soldiers as low physical fitness was found to be a stronger predictor of MSD in women than in men.^35^ The higher injury rate in women compared to men during BMT could actually be due to differences in average fitness levels.^20^ Thus, this modifiable risk factor should be addressed, especially since injury rates can decrease significantly as was shown in the US Army after the introduction of a mandatory physical training program for all BMT units.^36^

Taken together, our results confirm the importance of screening individuals before entering military training to implement primary and secondary prevention or rehabilitation strategies. Timely identification and early management of MSD are important prevention strategies.^37^ In addition, appropriate leadership and leadership awareness of injury prevention strategies are needed to effectively reduce MSD in military personnel.^38^ Furthermore, footwear and equipment modifications should also be considered as preventive strategies.^18,37,38^

It is important that individuals are physically prepared before entering BMT. A pre-training program, as the 12-week program including cardiorespiratory fitness and muscle strength training that Swedish recruits nowadays are encouraged to perform before BMT, is recommended. Equally important is the progressive increase of physical training and loading during BMT. To address these and other issues, the concept “Optimize training and exercise” as described in a handbook^39^ was currently adopted by the SwAF. The importance of leadership is stressed and a model for a progressive increase of physical loading based on carried load and the individual’s weight is described. The load should not exceed 34% of an individual’s BW during the first 12 weeks of training but can be progressively increased during the next 8 weeks, representing up to 47% of BW. A loading over 50% of BW should always be avoided. The MSP questionnaire and a test-battery with the overall aim to identify risk factors and early signs of MSD, low ratings of health, and insufficient physical performance are also described in the handbook.^39^ In addition, the significance of a relevant selection process certifying that set physical standards correctly reflect expected military job requirements is also addressed.

This study has some weaknesses that must be considered when interpreting the results. Data from the MSP questionnaire were self-reported and included retrospective information. This might have led to both an over- and underestimation of previous and present MSD. However, regarding results from our previous studies,^24–27^ we believe that the questionnaire can produce reliable data. Further, in comparison to medical records, self-reported MSD data are found to be accurate.^40^ Another limitation is the sample size, which made it impossible to make sub-group analyses and thereby investigate if the prevalence of MSD differed between e.g., military branches. Most importantly, the cross-sectional design that was employed cannot give information on causality. Thus, our identified associated factors cannot be seen as predictive risk factors as the design only gives evidence of associations. At the same time, the results corroborate previous findings in female military personnel,^20,22,23^ which strengthen our findings. However, we have not explored the situation for male recruits undergoing BMT and can therefore not know if our findings are sex specific or due to other factors. Future longitudinal studies are needed to confirm the results, to explore if the findings also apply to male recruits, and to evaluate the effectiveness of interventions addressing the identified modifiable factors.

## CONCLUSIONS

This study showed that the prevalence of MSD in Swedish female recruits was high before, during, and at the end of BMT, with knee and upper back as most frequent reported locations. Although the physical exposure during BMT was occasionally high, self-rated health was mainly perceived as good to excellent at the end of BMT. Previous MSD and being physically unprepared were related to MSD at the end of BMT. These important and relevant findings indicate the necessity for implementing interventions to increase physical fitness and for treatment of MSD at the beginning as well as during BMT.

## Data Availability

The datasets used and/or analyzed during the current study are available from the corresponding author on reasonable request.

## INSTITUTIONAL CLEARANCE

Does not apply.
